# Assessment of four different dietary amino acid profiles recommended for ISA Brown layer hens: A validation study

**DOI:** 10.1016/j.aninu.2024.01.009

**Published:** 2024-03-16

**Authors:** Mehdi Toghyani, Shemil Macelline, Juliano C. de Paula Dorigam, Peter V. Chrystal, Peter H. Selle, Sonia Y. Liu

**Affiliations:** a School of Life and Environmental Sciences, Faculty of Science, The University of Sydney, Camperdown 2006, NSW, Australia; b Poultry Research Foundation, The University of Sydney, Camden 2570, NSW, Australia; c Evonik Operations GmbH, Hanau-Wolfgang 63457, Germany

**Keywords:** Ideal amino acid profile, Regression model, Egg production, Egg mass

## Abstract

The current feeding study was designed to validate the two dietary essential amino acid profiles (EAAP) established based on linear broken-line (LBL) and quadratic broken-line (QBL) models, in a previous study, against Evonik (AMINOHen) and breeder recommendations for ISA Brown layers for peak production (PP, 20 to 44 weeks of age), and post peak production (post PP, 44 to 75 weeks of age). The EAAP based on LBL models on average had 19.5% and 26.0% lower digestible AA (Lys, Met + Cys, Thr, Trp, Ile and Val), than the EAAP based on QBL models for PP and post PP, respectively. The EAAP based on AMINOHen and breeder recommendation had lower digestible AA than QBL, and higher EAAP than LBL models for both production phases. At 20 weeks of age, 224 ISA Brown layer hens were weighed and randomly allocated to individual battery cages. Each of the four diets was replicated 8 times with 7 birds per replicate. Egg production was recorded daily, and egg weights were measured at the end of each week. Feed consumption was measured at the end of each period. The egg production rate was not significantly affected by the diets and remained at around 98.0% (PP) and 95.0% (post PP) (*P* > 0.05). Birds fed diets based on LBL recommendation consistently laid smaller eggs, resulting in a lower egg mass (59.8 vs. 62.0 g egg/hen per day during PP, and 60.3 vs. 63.0 g egg/hen per day during post PP; *P* < 0.05). Diets had no significant effect on feed intake and body weight (*P* > 0.05). The highest feed conversion ratio (FCR) during PP (*P* = 0.067) and post PP (*P* < 0.05) was recorded for the birds offered diets based on LBL recommendation. In conclusion, all four EAAP tested in this study support an above average egg production rate. However, the EAAP based on LBL models may potentially decrease the input feed cost per kilogram of eggs but are not set to optimise FCR and maximise egg mass.

## 1 Introduction

Protein, after energy, is the second most expensive nutrient in a layer hen's diet. Excess dietary crude protein can overload the gastrointestinal tract with excess AA and undigested protein (Apajalahti and Vienola, 2016), resulting in impaired feed efficiency, health and welfare issues as well as negative environmental impacts via excess nitrogen excretion. The dietary balance of AA significantly influences the efficiency of protein utilisation (Harms and Ivey, 1992). In order to precisely define the essential AA (EAA) requirements of birds for protein accretion and maintenance, the concept of “ideal EAA/protein” in nutrition was initially proposed in the 1950s by poultry nutritionists at the University of Illinois for broiler chicken diets (Fisher and Scott, 1954; Glista et al., 1951). By the late 1990s the ideal EAA ratios were adopted for swine (NRC, 1998), and meat chickens (Emmert and Baker, 1997; Mack et al., 1999), and eventually for layer hens (Bregendahl et al., 2008; Leeson and Summers, 2005; Rostagno et al., 2005).

In layer hens, in addition to the stage of production, egg size and egg mass output, a multitude of dietary, environmental, and genetic factors could also affect the actual AA requirements. However, the ideal EAA profile (EAAP) employs the concept that, whereas absolute AA requirements change due to genetic or environmental factors, the ratios amongst them are only slightly affected (Bregendahl et al., 2008). Furthermore, once an ideal ratio of EAA to Lys is established, then determining the Lys requirement accurately under a variety of conditions can assist in calculating the requirement for all other EAA by applying their ideal ratios to Lys (Mack et al., 1999). Precise feed formulation based on digestible AA and ideal AA profile aims to maximise protein deposition and minimise nitrogen output.

Dose-response models such as linear broken-line (LBL), quadratic broken-line (QBL) and quadratic polynomial (QP) are used to estimate the minimum concentrations of a nutrient that maximise a given outcome, thereby determining nutritional requirements for optimal performance (Gonçalves et al., 2016; Pesti et al., 2009; Vedenov and Pesti, 2008). Different statistical models predict different nutritional requirements (Gonçalves et al., 2016) where LBL models are more favourable for estimating AA requirements to calculate ideal AA ratios while curvilinear models such as exponential and quadratic models are better suited to establish the AA requirements for maximum performance (Mack et al., 1999). Kidd and Tillman (2016) suggested the slope of the AA response curve will begin to decrease at 70% to 80% of the maximum response because the needs of poorer performing birds begin to be met and the LBL model is likely to predict this early requirement (Liu et al., 2019); however, expression of responses from 90% to 100% of the maximum quadratic response represents the typical range in published reports to quantify a response and the use of the median level, 95% of the quadratic maximum, is used widely. Moreover, Pesti et al. (2009) concluded that despite the fact that non-linear models such as the saturation kinetics model, logistic model and a compartmental model may have the best statistical fit, they lack clear definition of nutritional requirements as required by nutritionists.

Recently, we determined the requirements of Lys, Met + Cys, Thr, Trp, Ile and Val for maximal egg mass in ISA Brown layers for peak and post peak production phases (Macelline et al., 2022). Data on egg production and egg mass were used to predict AA requirements by fitting LBL and QBL models, and then calculating the ratios relative to Lys. Overall, QBL models estimated higher AA requirements for egg mass and egg production rate than LBL models by 23% and 25%, respectively, which also resulted in two different ideal EAA ratios. The present feeding study was designed to validate the two dietary EAAP established based on LBL and QBL models against existing Evonik (AMINOHen) and breeder recommendations (Hendrix Genetics, 2020).

## 2 Materials and methods

### 2.1 Animal ethics statement

All experimental procedures were approved by the Research Integrity and Ethics Administration of the University of Sydney (2021/1883) and were in accordance with the Australian code for the care and use of animals for scientific purposes (National Health and Medical Research Council, 2013).

### 2.2 Experimental design and diets

Four recommended EAAP (Table 1) for peak (20 to 44 weeks of age or phase Ⅰ) and post peak (44 to 75 weeks of age or phase Ⅱ) production were used to formulate the experimental diets. The four dietary EAAP tested included: (1) and (2) the recommendation established by LBL and QBL models, respectively, from our previous studies (Macelline et al., 2022), (3) the recommendation by AMINOHen (Evonik) and (4) the recommendation by breeder (Hendrix Genetics, 2020).

**Table 1:** Dietary essential amino acid profiles and ideal ratios^1^ during the peak and post peak production.

Item, mg/hen per day	Lys	Met + Cys	Thr	Val	Ile	Trp
Peak of production (20 to 44 weeks of age)						
LBL models^2^	713 (100)	606 (85)	512 (72)	587 (82)	654 (92)	150 (21)
QBL models^2^	891 (100)	820 (92)	572 (64)	692 (78)	757 (85)	193 (22)
AMINOHen^3^	831 (100)	756 (91)	582 (70)	731 (88)	665 (80)	174 (21)
ISA Brown^4^	850 (100)	740 (87)	595 (70)	750 (88)	680 (80)	191 (22)
Post peak production (44 to 75 weeks of age)						
LBL models^2^	688 (100)	562 (82)	459 (67)	603 (88)	567 (82)	153 (22)
QBL models^2^	861 (100)	691 (80)	607 (70)	726 (84)	751 (87)	191 (22)
AMINOHen^3^	810 (100)	740 (91)	570 (70)	710 (88)	650 (80)	170 (21)
ISA Brown^4^	815 (100)	710 (87)	570 (70)	715 (88)	650 (80)	180 (22)

1 The ideal ratios (%) are shown in parentheses.

2 Established by linear broken-line (LBL) and quadratic broken-line (QBL) models from previous study (Macelline et al., 2022).

3 Recommended by Evonik AMINOHen.

4 Recommended by breeder (Hendrix Genetics, 2020).

Ingredient specifications were evaluated by near-infrared spectroscopy using the AMINOIR Advanced program (Evonik operations GmbH, Hanau, Germany) prior to feed formulation. The digestible AA values for each ingredient were used to formulate the diets. The diets were balanced for digestible Lys, Met + Cys, Thr, Ile, Val, and Trp. Accordingly, crystalline L-Lys HCl, DL-Met, L-Thr, L-Ile, L-Val and L-Trp were offered to all the diets at market prices (Table 2, Table 4). A minimum ratio of 105 was set for Arg across all the four diets, and the rest of the AA and crude protein were left to float in the formulations. Each diet was mixed separately and offered in mash form.

**Table 2:** Diet composition (as-fed basis) used in phase Ⅰ (peak production, 20 to 44 weeks of age).

Ingredients, %	LBL^1^	QBL^1^	AMINOHen^2^	Breeder^3^
Sorghum	37.40	29.25	32.55	31.13
Wheat	20.00	20.00	20.00	20.00
Soybean meal	11.50	18.75	15.75	17.00
Barley	9.00	9.00	9.00	9.00
Limestone grit	7.01	6.98	6.99	6.98
Canola meal	7.00	7.00	7.00	7.00
Canola oil	3.25	4.35	3.90	4.10
Limestone fine	2.50	2.50	2.50	2.50
Mono-dicalcium phosphate	0.85	0.80	0.85	0.85
NaHCO_3_	0.240	0.165	0.245	0.240
Salt	0.230	0.235	0.200	0.230
Lys-HCl	0.210	0.210	0.225	0.215
Vitamin-mineral premix^4^	0.200	0.200	0.200	0.200
DL-Met	0.180	0.330	0.290	0.265
Potassium carbonate	0.133	0.000	0.000	0.000
L-Ile	0.130	0.115	0.070	0.065
L-Thr	0.085	0.045	0.095	0.095
L-Val	0.000	0.000	0.055	0.050
Choline chloride	0.050	0.050	0.050	0.050
Carbohydrase^5^	0.020	0.020	0.020	0.020
Phytase^6^	0.007	0.007	0.007	0.007
Total	100.0	100.0	100.0	100.0
Total unbound AA	0.605	0.700	0.739	0.690
Diet cost, AUD/tonne	457.3	500.7	480.6	485.7

1 Established by linear broken-line (LBL) and quadratic broken-line (QBL) models from previous study (Macelline et al., 2022).

2 Recommended by Evonik AMINOHen.

3 Recommended by breeder (Hendrix Genetics, 2020).

4 Vitamin-mineral premix (per kilogram of air-dry diet): vitamin A 12,000 IU, vitamin D 33,000 IU, vitamin E 15 mg, vitamin K 2 mg, thiamine 2 mg, riboflavin 6 mg, pyridoxine 2 mg, calcium pantothenate 0.03 mg, folic acid 0.2 mg, niacin 45 mg, biotin 0.15 μg; calcium 0.5%, Co (as cobalt sulphate) 0.5 mg, Cu (as copper sulphate) 10 mg, iodine (as potassium iodine) 0.9 mg, iron (as ferrous sulphate) 80 mg, Mn (as manganous oxide) 80 mg, Se (as sodium selenite) 0.2 mg, Zn (as zinc oxide) 80 mg.

5 Axtra XB (Danisco).

6 Axtra Phy Gold (10,000 FTU, Danisco) to provide 700 FTU of phytase per kilogram of the diet.

**Table 3:** Diet composition (as-fed basis) used in phase Ⅱ (post peak production, 44 to 75 weeks of age).

Ingredients, %	LBL^1^	QBL^1^	AMINOHen^2^	Breeder^3^
Sorghum	40.23	33.24	34.60	34.12
Wheat	20.00	20.00	20.00	20.00
Barley	10.00	10.00	10.00	10.00
Soybean meal	8.29	14.37	13.28	13.77
Limestone grit	7.66	7.63	7.63	7.63
Canola meal	7.00	7.00	7.00	7.00
Canola oil	2.63	3.59	3.38	3.43
Limestone fine	2.00	2.00	2.00	2.00
Mono-dicalcium phosphate	0.88	0.84	0.85	0.85
NaHCO_3_	0.290	0.281	0.280	0.278
Salt	0.193	0.200	0.202	0.203
Lys-HCl	0.197	0.209	0.173	0.169
Vitamin-mineral premix^4^	0.200	0.200	0.200	0.200
DL-Met	0.115	0.174	0.221	0.195
Potassium carbonate	0.170	0.000	0.020	0.005
L-Ile	0.037	0.110	0.028	0.027
L-Thr	0.029	0.080	0.055	0.055
Choline chloride	0.050	0.050	0.050	0.050
Carbohydrase^5^	0.020	0.020	0.020	0.020
Phytase^6^	0.007	0.007	0.007	0.007
Total	100.0	100.0	100.0	100.0
Total unbound AA	0.378	0.573	0.477	0.446
Diet cost, AUD/tonne	417.3	468.3	448.0	449.4

1 Established by linear broken-line (LBL) and quadratic broken-line (QBL) models from previous study (Macelline et al., 2022).

2 Recommended by Evonik AMINOHen.

3 Recommended by breeder (Hendrix Genetics, 2020).

4 Vitamin-mineral premix (per kilogram of air-dry diet): vitamin A 12,000 IU, vitamin D 33,000 IU, vitamin E 15 mg, vitamin K 2 mg, thiamine 2 mg, riboflavin 6 mg, pyridoxine 2 mg, calcium pantothenate 0.03 mg, folic acid 0.2 mg, niacin 45 mg, biotin 0.15 μg; calcium 0.5%, Co (as cobalt sulphate) 0.5 mg, Cu (as copper sulphate) 10 mg, iodine (as potassium iodine) 0.9 mg, iron (as ferrous sulphate) 80 mg, Mn (as manganous oxide) 80 mg, Se (as sodium selenite) 0.2 mg, Zn (as zinc oxide) 80 mg.

5 Axtra XB (Danisco).

6 Axtra Phy Gold (10,000 FTU, Danisco) to provide 700 FTU of phytase per kg of the diet.

### 2.3 Birds and housing

A total of 224 ISA Brown pullets at 16 weeks of age were allocated to individual battery cages (wired-bottom) equipped with an individual nipple drinker and trough feeder. The birds were fed a pre-lay diet for 10 d, and then the pre-lay diet was replaced by a pre-peaking diet formulated to the specifications recommended by the breeder (Hendrix Genetics, 2020). During this period, birds were monitored for egg production and those that were not laying were replaced. At week 20 of age, all the birds were weighed and randomly allocated to their respective dietary treatments to have an average body weight of (1860 ± 10) g across treatments. Each diet was replicated 8 times with 7 birds per replicate unit. Birds were kept in an environmentally controlled layer shed where temperature, lighting, and ventilation were set according to ISA Brown Management guidelines (Hendrix Genetics, 2020).

### 2.4 Data collection and chemical analysis

The experimental diets were offered ad-libitum. Eggs were collected daily; egg production was calculated as a function of the number of birds, corrected for mortality, and egg weights were measured at the end of each week from eggs laid within 48 h prior to weighing. Feed consumption was measured at the end of each period on a replicate basis.

Egg production data and egg weights were used to calculate egg mass in each replicate. Feed conversion ratio (FCR) was defined as the feed intake required to produce 1 g of egg mass. Hen-day egg production (HDEP) was calculated using number of hen-days from 20 to 75 weeks and the number of eggs laid during the same period. Diet cost (AUD/kg) and FCR for each treatment were used to calculate feed cost (cents) per kilogram of eggs for peak and post peak production.

Birds were individually weighed at the beginning of the study and at the end of each phase. At the end of the study, 2 birds per replicate were randomly selected and euthanized. The abdominal fat pad was removed, individually weighed and recorded as gram per 100 g of live body weight. The femur and tibia bones were also removed and used to measure their breaking strength and ash percentage.

Egg and eggshell quality traits (yolk weight, albumen weight, albumen height, Haugh unit [HU], eggshell breaking strength, and shell weight) were determined using the eggs collected at week 44 and 75 of age. In total, 30% of the eggs were used to measure external and internal egg quality parameters at each time point.

First eggs were weighed, and then eggshell breaking strength (N/cm^2^) was determined at the broad end of the eggs using a 3-point bending test of the peak force to fracture using a texture analyzer (Perten TVT 6700, Stockholm, Sweden), fitted with a cylindrical probe 75 mm in diameter. Then the eggs were carefully broken out onto a flat, level glass surface on a metal stand located above a reflective mirror for internal egg assessment. An albumen height gauge (Technical Services and Supplies, York, United Kingdom) was used to measure the height of the thick albumen. The HU was then calculated as HU = 100 × log [(*h* + 7.57) − (1.7 × *w*^0.37^)], where *h* (mm) = albumen height, *w* (g) = egg weight (Monira et al., 2003). The albumen and yolk were carefully separated using a plastic scrapper. They were both weighed and expressed as percentage of whole egg weight. The eggshell membranes were removed, then the shells were washed and air dried. The weight of the air-dried shells was recorded and expressed as a percentage of the weight of the whole egg.

The right tibiae and femurs were removed and then the breaking strength (N/cm^2^) was determined as the peak force to fracture at the mid shaft using a texture analyzer (Perten TVT 6700), fitted with a breaking probe. All bones were held in the same orientation and the force was applied at the mid-length of the bone. The broken tibiae and femurs were used to determine the ash content. For this the bones were dried at 105 °C for 24 h then ignited to ash at 600 °C for 8 h, cooled in a desiccator and weighed. The percentage of ash was determined relative to the dry weight.

Triplicate representative composite samples from each diet (as-fed basis) were collected and analyzed for mineral concentration using an inductively coupled plasma-optical emission spectrometer (ICP-OES) (Agilent, Mulgrave, Victoria, Australia) following the methodology described by Maxfield and Mindak (1985). Amino acid concentrations in the diets were determined by 24-h liquid hydrolysis at 110 °C in 6 mol/L HCl and then AA were analyzed by ultra-performance liquid chromatography (Waters, Milford, MA, USA) (Boogers et al., 2008). The analyzed total AA concentrations in diets for phase Ⅰ and Ⅱ of the study are presented in Table 3, Table 5, respectively. The nitrogen contents of raw ingredients and diet samples were determined on a 0.25-g sample in a combustion analyzer (FP-2000, LECO Corp., St Joseph, MI, USA) using ethylenediaminetetraacetic acid (EDTA) as a calibration standard, with crude protein being calculated by multiplying percentage nitrogen by a correction factor (6.25) as described by Siriwan et al. (1993). Starch concentrations of the diets were determined by a procedure based on dimethyl sulfoxide, α-amylase and amyloglucosidase, as described in Mahasukhonthachat et al. (2010).

**Table 4:** Nutritional composition of diets (as-fed basis) used in phase Ⅰ (peak production, 20 to 44 weeks of age).

Nutrients^1^	LBL^2^	QBL^2^	AMINOHen^3^	Breeder^4^
AME, kcal/kg	2800	2800	2800	2800
NE, kcal/kg	2242	2238	2239	2240
Crude protein	14.8 (15.1)	17.4 (17.1)	16.4 (16.4)	16.8 (16.7)
Lys	0.80 (0.75)	0.99 (0.88)	0.93 (0.81)	0.95 (0.88)
Met	0.41 (0.37)	0.59 (0.53)	0.54 (0.46)	0.52 (0.45)
Met + Cys	0.68 (0.62)	0.90 (0.80)	0.83 (0.71)	0.82 (0.71)
Thr	0.61 (0.59)	0.68 (0.63)	0.68 (0.63)	0.70 (0.66)
Ile	0.73 (0.73)	0.84 (0.78)	0.75 (0.69)	0.76 (0.71)
Leu	1.27 (1.42)	1.43 (1.42)	1.36 (1.41)	1.39 (1.39)
Trp	0.19 (0.17)	0.23 (0.21)	0.21 (0.19)	0.22 (0.19)
Arg	0.84 (0.81)	1.05 (0.96)	0.96 (0.89)	1.00 (0.93)
His	0.36 (0.36)	0.43 (0.40)	0.40 (0.38)	0.41 (0.39)
Val	0.71 (0.73)	0.84 (0.79)	0.84 (0.79)	0.86 (0.80)
Dig Lys	0.72	0.89	0.83	0.85
Dig Met	0.38	0.56	0.51	0.49
Dig Met + Cys	0.61	0.82	0.76	0.74
Dig Thr	0.52	0.57	0.58	0.60
Dig Ile	0.66	0.76	0.67	0.68
Dig Trp	0.16	0.20	0.18	0.19
Dig Arg	0.76	0.96	0.87	0.91
Dig His	0.32	0.38	0.36	0.37
Dig Val	0.62	0.72	0.73	0.75
Crude fat	5.18	6.13	5.74	5.90
Crude fibre	2.95	3.10	3.03	3.06
Starch	44.8 (42.0)	39.0 (38.1)	41.4 (40.3)	40.4 (39.5)
Calcium	4.00 (4.15)	4.00 (4.21)	4.00 (4.23)	4.00 (4.20)
Total phosphorus	0.53 (0.63)	0.55 (0.64)	0.54 (0.66)	0.55 (0.63)
Available phosphorus	0.45	0.45	0.45	0.45
Ash	13.0	13.1	13.0	13.1
Sodium	0.20 (0.17)	0.18 (0.16)	0.19 (0.18)	0.20 (0.18)
Chloride	0.24	0.24	0.22	0.24
Potassium	0.65 (0.71)	0.70 (0.75)	0.65 (0.72)	0.67 (0.74)
Electrolyte balance, mEq/kg	186	190	185	190

Dig = digestible.

1 Analyzed values are presented in parenthesis. Values are expressed in %, unless stated otherwise.

2 Established by linear broken-line (LBL) and quadratic broken-line (QBL) models from previous study (Macelline et al., 2022).

3 Recommended by Evonik AMINOHen.

4 Recommended by breeder (Hendrix Genetics, 2020).

**Table 5:** Nutritional composition of diets (as-fed basis) used in phase Ⅱ (post peak production, 44 to 75 weeks of age).

Nutrient^1^	LBL^2^	QBL^2^	AMINOHen®^3^	Breeder^4^
AME, kcal/kg	2780	2780	2780	2780
NE, kcal/kg	2226	2224	2224	2224
Crude protein	13.6 (14.1)	15.8 (15.8)	15.4 (15.3)	15.6 (15.9)
Lys	0.71 (0.71)	0.87 (0.81)	0.82 (0.80)	0.83 (0.78)
Met	0.33 (0.32)	0.42 (0.38)	0.46 (0.45)	0.43 (0.40)
Met + Cys	0.58 (0.55)	0.70 (0.63)	0.73 (0.71)	0.71 (0.65)
Thr	0.51 (0.52)	0.65 (0.62)	0.61 (0.63)	0.61 (0.59)
Ile	0.58 (0.60)	0.76 (0.74)	0.66 (0.68)	0.67 (0.65)
Leu	1.22 (1.28)	1.35 (1.38)	1.33 (1.37)	1.34 (1.33)
Trp	0.17 (0.16)	0.20 (0.17)	0.20 (0.18)	0.20 (0.18)
Arg	0.74 (0.73)	0.92 (0.86)	0.89 (0.90)	0.90 (0.86)
His	0.34 (0.33)	0.39 (0.38)	0.38 (0.39)	0.39 (0.37)
Val	0.66 (0.67)	0.76 (0.75)	0.74 (0.76)	0.75 (0.73)
Dig Lys	0.63	0.78	0.73	0.74
Dig Met	0.31	0.39	0.43	0.41
Dig Met + Cys	0.52	0.63	0.67	0.64
Dig Thr	0.43	0.55	0.51	0.52
Dig Ile	0.52	0.68	0.59	0.59
Dig Trp	0.15	0.17	0.17	0.17
Dig Arg	0.67	0.83	0.80	0.82
Dig His	0.29	0.34	0.33	0.34
Dig Val	0.57	0.66	0.64	0.65
Crude fat	4.66	5.47	5.29	5.33
Crude fibre	2.92	3.04	3.02	3.03
Starch	47.2 (45.3)	42.3 (43.1)	43.3 (42.4)	43.0 (43.6)
Calcium	4.05 (4.22)	4.05 (4.36)	4.05 (4.20)	4.05 (4.28)
Total phosphorus	0.53 (0.58)	0.54 (0.58)	0.54 (0.61)	0.54 (0.60)
Available phosphorus	0.45	0.45	0.45	0.45
Ash	13.2	13.2	13.2	13.2
Sodium	0.20 (0.17)	0.20 (0.16)	0.20 (0.17)	0.20 (0.18)
Chloride	0.22	0.22	0.22	0.22
Potassium	0.62 (0.68)	0.63 (0.71)	0.62 (0.66)	0.62 (0.69)
Electrolyte balance, mEq/kg	185	185	185	185

Dig = digestible.

1 Analyzed values are presented in parentheses. Values are expressed in %, unless stated otherwise.

2 Established by linear broken-line (LBL) and quadratic broken-line (QBL) models from previous study (Macelline et al., 2022).

3 Recommended by Evonik AMINOHen.

4 Recommended by breeder (Hendrix Genetics, 2020).

### 2.5 Statistical analysis

All the data collected were checked for normal distribution prior to conducting statistical analysis. Each replicate (7 cages) was considered as an experimental unit, and the values presented in the tables are means with a pooled standard error of the mean (SEM) (*n* = 32). Data were subjected to one-way ANOVA analysis as a completely randomized design using the General Linear Model procedure of JMP Pro 16. If a significant effect of treatment was detected, differences between treatments were separated by Tukey's Honest Significant Difference (HSD) test. Significance was set at *P* < 0.05.

## 3 Results

The analysed nutrients of the diets are presented in Table 4, Table 5 for phase Ⅰ and Ⅱ of the study, respectively. The analysed crude protein content of the diets was very close to the calculated values. The analysed total calcium and phosphorus contents were higher than the calculated values for all the four diets in both phases. The analysed AA concentrations of the diets for some AA were lower than the calculated values. However, the deviation followed the same trend amongst all the four diets for both phases, and yet the expected differences in AA profile of the diets were detectable. This variation to some extent could be explained by lab analytical tolerances as the diets were prepared in mash form, and some level of segregation, especially for the synthetic AA, could have occurred during sample collection and preparation.

The overall mortality until week 75 in this trial was 3.5% and was not impacted by dietary treatments (*P* > 0.05). The data on production performance, feed intake, FCR, body weight and feed cost per kilogram of eggs for peak production (20 to 44 weeks of age) and post peak production (44 to 75 weeks of age) are presented in Table 6, Table 7, respectively. The differences in dietary AA profiles did not have any significant effect on egg production rate (%) over both periods (*P* > 0.05). However, birds offered diets with an AA profile based on LBL models recorded the lowest egg weight at peak (3.5% lower) and post peak (4.5% lower) production (*P* < 0.05), and as a result had the lowest egg mass (*P* < 0.05) than the other three treatments. Feed conversion ratio tended (*P* = 0.067) to be higher for the birds in the LBL group from 20 to 44 weeks of age (2.08 vs. an average of 1.98) and was the highest from 44 to 75 weeks of age (2.09 vs. an average of 1.97; *P* < 0.05). There was no treatment difference on feed intake, body weight gain and feed cost per kilogram of egg (*P* > 0.05). Table 8 presents data on laying performance and body weight gain over the entire production period (20 to 75 weeks of age). Similar to the results observed for peak and post peak production, diets based on LBL profile had an overall lower egg mass and higher FCR (*P* < 0.05) than the other three treatments, but feed intake, egg production rate, HDEP and body weight gain were not impacted by differences in dietary AA profile (*P* > 0.05).

**Table 6:** Production performance and body weight gain in laying hens during peak production (20 to 44 weeks of age)^1^.

Dietary essential amino acid profile	Egg weight, g/egg	Egg production rate, %	Egg mass, g egg/hen	Feed intake, g/hen per day	FCR, g/g	BW (week 20), g/hen	BW (week 44), g/hen	BWG (week 20 to 44), g/hen	Feed cost, cent/kg egg
LBL models^2^	61.1^b^	97.9	59.8^b^	124.3	2.079	1866	2258	392	95.06
QBL models^2^	63.3^a^	98.3	62.3^a^	122.1	1.960	1859	2314	454	98.15
AMINOHen^3^	63.4^a^	98.4	62.4^a^	123.8	1.984	1862	2297	434	95.33
ISA Brown^4^	62.8^a^	98.2	61.6^a^	122.9	1.995	1869	2292	423	96.87
SEM	0.48	0.40	0.53	1.51	0.0310	6.6	26.6	24.4	1.529
*P*-value	0.006	0.833	0.006	0.728	0.067	0.759	0.510	0.341	0.459

FCR = feed conversion ratio; BW = body weight; BWG = body weight gain.

^a–b^Values in a column with no common superscripts differ significantly (*P* < 0.05).

1 Mean values are based on 7 layers per replicate and 8 replicates per treatment.

2 Established by linear broken-line (LBL) and quadratic broken-line (QBL) models from previous study (Macelline et al., 2022).

3 Recommended by Evonik AMINOHen.

4 Recommended by breeder (Hendrix Genetics, 2020).

**Table 7:** Production performance and body weight gain in laying hens during post peak production (44 to 75 weeks of age)^1^.

Dietary essential amino acid profile	Egg weight, g/egg	Egg production rate, %	Egg mass, g egg/hen	Feed intake, g/hen per day	FCR, g/g	BW (week 44), g/hen	BW (week 75), g/hen	BWG (week 44 to 75), g/hen	Feed cost, cent/kg egg
LBL models^2^	63.3^b^	95.2	60.3^b^	126.0	2.092^a^	2258	2345	87.6	87.28
QBL models^2^	66.4^a^	95.1	63.1^a^	123.0	1.948^b^	2314	2403	88.9	91.26
AMINOHen^3^	66.3^a^	95.9	63.5^a^	125.9	1.981^b^	2297	2411	113.8	88.78
ISA Brown^4^	65.3^a^	95.2	62.2^ab^	123.1	1.979^b^	2292	2410	118.1	88.97
SEM	0.64	0.61	0.66	1.74	0.0260	26.6	28.9	19.80	1.157
*P*-value	0.006	0.784	0.007	0.439	0.003	0.510	0.332	0.585	0.134

FCR = feed conversion ratio; BW = body weight; BWG = body weight gain.

^a–b^Values in a column with no common superscripts differ significantly (*P* < 0.05).

1 Mean values are based on 7 layers per replicate and 8 replicates per treatment.

2 Established by linear broken-line (LBL) and quadratic broken-line (QBL) models from previous study (Macelline et al., 2022).

3 Recommended by Evonik AMINOHen.

4 Recommended by breeder (Hendrix Genetics, 2020).

**Table 8:** Production performance and body weight gain in laying hens during entire production period (20 to 75 weeks of age)^1^.

Dietary essential amino acid profile	Egg weight, g/egg	Egg production rate, %	Egg mass, g egg/hen	HDEP, egg/hen	Feed intake, g/hen per day	FCR, g/g	BWG (week 20 to 75), g/hen	Feed cost, cent/kg egg
LBL models^2^	62.3^b^	96.4	60.1^b^	371.3	125.2	2.085^a^	480	90.82
QBL models^2^	65.0^a^	96.5	62.7^a^	371.7	122.6	1.954^b^	544	94.38
AMINOHen^3^	65.0^a^	97.0	63.0^a^	373.5	125.0	1.982^b^	548	91.75
ISA Brown^4^	64.2^a^	96.6	62.0^a^	371.8	123.0	1.986^b^	541	92.54
SEM	0.54	0.46	0.55	1.77	1.43	0.0250	29.9	1.117
*P*-value	0.004	0.812	0.003	0.812	0.467	0.006	0.245	0.200

HDEP = hen-day egg production; FCR = feed conversion ratio; BWG = body weight gain.

^a–b^Values in a column with no common superscripts differ significantly (*P* < 0.05).

1 Mean values are based on 7 layers per replicate and 8 replicates per treatment.

2 Established by linear broken-line (LBL) and quadratic broken-line (QBL) models from previous study (Macelline et al., 2022).

3 Recommended by Evonik AMINOHen.

4 Recommended by breeder (Hendrix Genetics, 2020).

According to the data presented in Table 9, except the relative egg yolk weight at week 44, which was higher in birds fed diets with dietary AA profile based on the LBL models and breeder recommendation (*P* < 0.05), the other parameters measured including eggshell breaking strength, relative shell weight, relative albumin weight and HU were not influenced by the dietary treatments (*P* > 0.05).

**Table 9:** Egg quality parameters determined at week 44 and **75**^1^.

Dietary essential amino acid profile	Egg shell breaking strength, N/cm^2^		Shell percentage, g/100 g eggs		Albumin percentage, g/100 g eggs		Yolk percentage,g/100 g eggs		Haugh unit	
	week 44	week 75	week 44	week 75	week 44	week 75	week 44	week 75	week 44	week 75
LBL models^2^	48.2	41.5	14.5	13.7	58.7	55.8	26.7^a^	30.5	94.2	95.7
QBL models^2^	47.7	44.1	14.7	13.2	59.6	56.6	25.8^ab^	30.2	97.0	91.0
AMINOHen^3^	47.3	43.7	13.6	14.1	61.5	57.2	24.8^b^	28.6	97.5	92.5
ISA Brown^4^	41.2	41.2	14.1	13.3	59.8	57.2	26.1^a^	29.5	95.0	94.5
SEM	1.94	1.75	0.51	0.30	0.75	0.84	0.43	0.77	1.54	4.46
*P*-value	0.102	0.548	0.479	0.150	0.102	0.567	0.038	0.329	0.393	0.885

^a–b^Values in a column with no common superscripts differ significantly (*P* < 0.05).

1 Mean values are based on 2 eggs per replicate and 8 replicates per treatment at each time point.

2 Established by linear broken-line (LBL) and quadratic broken-line (QBL) models from previous study (Macelline et al., 2022).

3 Recommended by Evonik AMINOHen.

4 Recommended by breeder (Hendrix Genetics, 2020).

The treatments had no significant effect on relative abdominal fat pad weight, tibia and femur breaking strength and ash percentage (Table 10; *P* > 0.05).

**Table 10:** Breaking strength (N/cm^2^) and ash (%) of tibia and femur and abdominal fat pad weight (g/100 g body weight) determined at week 75^1^.

Dietary essential amino acid profile	Femur		Tibia		Fat pad
	Breaking strength	Ash	Breaking strength	Ash	
LBL models^2^	239	33.0	259	30.9	7.01
QBL models^2^	252	32.6	254	31.0	6.62
AMINOHen^3^	241	33.5	249	31.3	6.31
ISA Brown^4^	245	33.0	262	31.7	6.21
SEM	8.9	0.97	6.4	0.88	0.320
*P*-value	0.723	0.920	0.951	0.950	0.334

1 Mean values are based on 2 layers per replicate and 8 replicates per treatment.

2 Established by linear broken-line (LBL) and quadratic broken-line (QBL) models from previous study (Macelline et al., 2022).

3 Recommended by Evonik AMINOHen.

4 Recommended by breeder (Hendrix Genetics, 2020).

## 4 Discussion

In modern laying hens, the extensive genetic selection and breeding programs coupled with advanced nutritional strategies have improved birds' productivity in terms of egg production rate, egg mass, and feed efficiency (Kumar et al., 2018). Such changes have made these birds more responsive and simultaneously susceptible to dietary interventions. Thus, even marginal deficiency of EAA or amounts in excess of actual requirements are stressful to birds, and negatively influence productivity and increase production cost and carbon footprint. Matching the EAAP of the diet as closely as possible to the profile required by the birds at a certain stage of production is crucial for maximising laying and economic performance.

For all the treatments in this experiment, the egg production rate (96.5% vs. 93.7%) and the HDEP (371 vs. 361 egg/hen; 20 to 75 weeks of age) was superior to the breeder's performance objective (Hendrix Genetics, 2020), and not impacted by different EAAP in the diet. However, hens in this study gained more weight and had higher feed intake per day compared to the breeder performance objectives (124 vs. 112 g/d) but except LBL group (2.09) the rest of the groups exhibited better FCR than the breeder guidelines (1.97 vs. 2.07). These results suggest that the EAA content of the diets for maximal egg production rate were not limited within any of the four dietary EAAP tested in this study. However, it is worth noting that the interpretations of these findings are well-grounded within the experimental settings and context of this trial, where feed intake was not a limiting factor. Under commercial settings and different production systems the predicted feed intake should be taken into consideration while revising dietary EAAP to optimise egg production rate.

The most noticeable differences across the different EAAP tested in this validation study were between LBL and QBL treatments. The LBL diets for phase Ⅰ had approximately 25%, 35%, 11%, 16% and 28% less digestible Lys, Met + Cys, Thr, Ile and Trp content, respectively, compared to QBL diets. For phase Ⅱ, LBL diets on average had 26% lower digestible EAA content, with the biggest difference for Thr and Ile, which was close to 33% lower than the QBL diets. This also resulted in differences for ratios of EAA to Lys. For example, for phase Ⅰ, the ratios of Met + Cys and Trp to Lys were 8.2% and 4.7% higher, respectively, in the QBL diet than the LBL diet, while the LBL diet had higher ratios for Thr (11.1%), Val (4.8%) and Ile (7.6%). For phase Ⅱ, the magnitude of differences was smaller with opposite trends to phase Ⅰ, where the QBL diet had lower ratios for Met + Cys (2.5%), and Val (4.5%), and higher ratios for Thr (4.5%) and Ile (6.1%) than the LBL diet. The ratios based on breeder recommendation and AMINOHen did not change from phase Ⅰ to Ⅱ. Increasing dietary Lys has been shown to improve egg production, egg weight, egg mass, and feed conversion efficiency in laying hens (Faria et al., 2003; Kakhki et al., 2016). In the present study, the reduced egg weight and increased FCR in LBL treatment could have partially been caused by the lower crude protein and Lys content of the LBL diets. However, as the LBL diet was balanced based on the ideal EAA ratio the lower dietary crude protein and Lys per se did not compromise egg production rate. Similarly, Spangler et al. (2019) and Kumar et al. (2018) showed that laying hens required higher digestible Lys to optimise egg mass and the FCR in comparison to egg production.

Met and Cys, the total sulphur-containing AA (TSAA), have long been recognized as the first limiting AA in practical poultry diets (Schutte and Van Weerden, 1978). Met intake has been reported to regulate feed intake and also egg size (Carvalho et al., 2018). However, excess dietary Met concentrations have been shown to negatively impact on feed intake despite the overall increased Met intake (Shafer et al., 1998). In phase Ⅰ of the present study, the lowest dietary digestible Met + Cys was 0.61% in the LBL diet while the QBL diet had the highest content of 0.82%. In phase Ⅱ, the LBL diet had approximately 25% lower digestible Met + Cys compared to the other dietary treatments. Interestingly, the differences in EAA of the diets, particularly Met + Cys content, did not affect feed intake and egg production rate in either phase. However, birds fed LBL diets consistently laid smaller eggs, while the extra Met + Cys in the QBL diet did not necessarily lead to higher egg weight compared to diets formulated to AMINOHen and breeder EAAP, indicating that Met + Cys content in QBL diets could have already reached the maximum threshold where egg weight responds to Met + Cys, and furthermore the TSAA content has not been the only driver of egg weight. However, apart from total digestible Lys, the only difference between AMINOHen and breeder recommendation was the higher Met + Cys content and ratio to Lys in diets based on AMINOHen compared to breeder recommendation, which numerically resulted in higher egg weight by 1.25% or 0.8 g/egg. These results confirm the earlier reports indicating that for each 0.05% increase in Met + Cys above 0.23%, the egg weight increases by 0.7 g (Calderon and Jensen, 1990; Waldroup and Hellwig, 1995).

Thr is the third limiting AA in conventional poultry diets (Fernandez et al., 1994; Kidd et al., 2013). The importance of Thr in poultry is because of its prominent role in intestinal mucin secretion and in production of antibodies (Cardoso et al., 2014), and hence, its role in immunity. Despite the higher ratio of Thr to Lys in the LBL diet in phase Ⅰ, the digestible content of Thr in this diet was approximately 11.0% lower than the other three diets, and in phase Ⅱ both the ratio and absolute values were lower in the LBL diet. The effects of Thr intake on feed intake reported in the literature are inconsistent. However, an intake of less than 420 mg/bird per day has been reported to reduce feed intake (Bregendahl et al., 2008; Faria et al., 2003). The calculated digestible Thr intake of 646 and 542 mg/hen per day in phase Ⅰ and Ⅱ, respectively, in birds fed the diets with lowest Thr (LBL diet) was well above the minimum Thr intake to compromise feed intake. However, it is worth noting that Thr requirement may be higher under gastrointestinal challenge, disease conditions and different production systems, i.e., free range and/or barn (Azzam et al., 2011).

Trp is the precursor of several metabolites, including serotonin, indoleamine 2,3-dioxygenase, and quinolinic acid (Bai et al., 2017; Khattak and Helmbrecht, 2019). Therefore, Trp is involved in different roles and functions in the body such as reducing stress, increasing appetite (Le Floc'h and Seve, 2007), maintaining immune functions (Bai et al., 2017) and metabolism of carbohydrates and lipids (Khattak and Helmbrecht, 2019) and protein synthesis (Corzo et al., 2005). Although, Trp to Lys ratio was the least variable ratio across the four diets, but the total digestible Trp of the LBL diet was lower by 28.0% and 25.0% compared to the average values of the other three diets for phase Ⅰ and Ⅱ, respectively. Dietary Trp levels below 156 mg/kg have been reported to depress feed intake (Mousavi et al., 2018; Wen et al., 2019). The calculated digestible Trp intake of the birds fed diets based on LBL models, with the lowest Trp content, was close to 200 and 190 mg/hen per day in phase Ⅰ and Ⅱ, respectively, which should have not impacted feed intake. Moreover, dietary Trp levels have been reported not to have any significant effect on egg size (Mousavi et al., 2018).

Because of the antagonisms that exist between branch-chained AA (Ile, Val and Leu), adjusting the levels of these AA, particularly Ile and Val, are important in practical layer diets (Azzam et al., 2015; Peganova and Eder, 2002). Both Ile and Val are involved in fatty acid metabolism in the liver (Bai et al., 2015), and as such their deficiency or disparity may compromise hepatic yolk-lipoprotein production and consequently egg production. In a recent review on AA requirements of laying hens, Macelline et al. (2022) detected a positive correlation between dietary Val levels and feed intake, while no correlations were found between dietary Ile levels and feed intake. The differences in Ile and Val content across the four dietary EAAP tested in this study did not affect egg production rate or feed intake. However, the lower Ile and Val content of the diets based on LBL models could have partly contributed to the lower egg size in this group.

All the diets in this study were designed to be least-cost formulas based on ingredient market prices at the time of formulation. Therefore, the dynamics of the diets, particularly the inclusion levels of supplemental fat and synthetic AA were different amongst these diets. Diets formulated based on LBL models had lower fat inclusion and total unbound or synthetic AA. Studies on broiler chickens have shown that the digestive dynamics of protein-bound and unbound AA are inherently different in poultry, which also impacts their performance, particularly in reduced crude protein diets (Liu and Selle, 2017; Selle and Liu, 2019). Selle and Liu (2019) describe the concept of protein digestive dynamics as a combination of protein digestion rates, absorption of AA, and their transition across the gut mucosa into the portal circulation. However, this concept has yet to be fully explored and evaluated in laying hens. Earlier work by Sohail et al. (2002), demonstrated the beneficial effect of including unbound Lys, Thr, Ile and Trp on egg production rate and egg mass, irrespective of dietary crude protein levels. Findings of very recent research on reduced crude protein diets for laying hens showed that reduction of the crude protein content of diet from 16.1% to 14.7%, with similar content of digestible EAA, resulted in a higher laying rate and lower egg weight. However, further reduction of crude protein content to 13.5% and 12.0% impaired the laying rate, egg weight, egg mass, and feed efficiency (Van Harn et al., 2021). It would be very interesting and yet difficult to ascertain the plausible confounding effect(s) of differences in dietary crude protein, crude fat and unbound AA levels on the results obtained in the current study. Nevertheless, the results reported herein indicate that reduction of 2.2 (phase Ⅰ) and 2.6 (phase Ⅱ) percentage units in crude protein (LBL vs. QBL diets), when diets are balanced based on different EAAP, does not compromise egg production rate and feed intake, although may to some extent explain the higher FCR and lower egg weights in hens fed diets based on LBL profile.

Historically, egg numbers and eggshell breaking strength have a negative correlation with egg weight (Hy-Line 2022). However, despite the differences in egg weights observed in this study in response to diets formulated based on different EAAP, the eggshell breaking strength was not affected. Birds fed the diets based on LBL models numerically had the highest abdominal fat percentage, approximately 7.0% higher compared to other treatments. Although the energy expenditure and retention in layer hens is markedly different to meat-chickens, lowering dietary crude protein in meat-chicken diets has always been associated with higher abdominal fat yield (Chrystal et al., 2021; Cowieson et al., 2020). The diets based on LBL models had the lowest crude protein content for both phase Ⅰ and Ⅱ, which could explain the differences in abdominal fat pad observed in this study. In addition, lowering dietary crude protein has been reported to compromise tibia bone integrity and breaking strength in meat-chickens (Cowieson et al., 2020). However, tibia and femur bone breaking strength and ash percentage were not impacted by the differences in EAAP and/or dietary crude protein levels in this study. This is probably due to the fact that the nutrient balance of the diets fed during the rearing period (0 to 16 weeks) has a greater impact on layer hens' bone and overall skeletal integrity than the during laying period, provided the mineral balance, particularly calcium and phosphorous, of the layer hen diet is not a limiting factor.

Adjusting dietary nutrient levels based on the QBL models follows the law of diminishing returns (Wang et al., 2019). QBL models predict nutritional responses over a wide range of nutrient input and are more accurate in describing the biological responses than the LBL models where the biological responses are characterized with variation between the individuals of the population being measured (Mercer, 1980). Overall, the results obtained in this validation trial further attest that the AA requirements determined with the LBL model can be used for obtaining the ideal ratios among EAA, whereas QBL models are better suited to establish the AA requirements for maximum performance response(s). Previous studies have also indicated that broken-line regression method results in lower AA requirements than when a non-linear curve fitting is applied to the same dataset (Bregendahl et al., 2008). Similarly, in calcium requirement studies for grower and finisher broiler chicks, lower calcium requirements were estimated with LBL models compared to QBL (Walk et al., 2022b, Walk et al., 2022a).

## 5 Conclusion

There is no doubt that the balance of AA in laying hen diets is an important nutritional variable that affects the economic efficiency of an egg laying operation. The four dietary EAAP tested in this validation trial supported a superior egg production rate without compromising internal and external egg quality parameters, or bone integrity. The EAAP based on QBL regression equations could be used to maximise egg mass and optimise FCR but not the feed cost per kilogram of egg. On the other hand, the EAAP based on LBL models are not set to maximise egg weight and egg mass but could potentially optimise feed cost per kilogram of egg without compromising egg production rate, depending on feed ingredient prices, particularly soybean meal and supplemental fat. The AMINOHen and breeder profiles are better set to balance both egg mass output and the feed cost per kilogram of egg. The data generated in this validation study could be used to formulate practical diets for ISA Brown layer hens in an attempt to optimise production efficiency and economics for specific market conditions.

## Author contributions

All six authors contributed towards the completion of this study and have read and approved this manuscript. **Mehdi Toghyani** and **Sonia Yun Liu** were the principal investigators of the relevant project. **Mehdi Toghyani** formulated the diets and drafted the initial manuscript. **Shemil Macelline** and **Mehdi Toghyani** conducted and supervised the feeding study. **Peter Vincent Chrystal** and **Juliano C. de Paula Dorigam** were involved in the conceptualization and methodology. **Peter Henry Selle** and **Shemil Macelline** completed the statistical analyses. All authors contributed to further editing and review of the manuscript. **Mehdi Toghyani** was responsible for the final editing and submission of the manuscript.

## Declaration of competing interest

We declare that we have no financial and personal relationships with other people or organizations that can inappropriately influence our work, and there is no professional or other personal interest of any nature or kind in any product, service and/or company that could be construed as influencing the content of this paper. Despite one of the co-authors (Juliano C.de Paula Dorigam) being employed by Evonik, the company that co-funded this project, there are no conflicts of interest and Juliano’s involvement in this research was transparent and does not compromise the integrity or impartiality of the findings.
